# Characterization of the Clinical Significance and Immunological Landscapes of a Novel TMEMs Signature in Hepatocellular Carcinoma and the Contribution of TMEM201 to Hepatocarcinogenesis

**DOI:** 10.3390/ijms241210285

**Published:** 2023-06-17

**Authors:** Desheng Chen, Yichao Lou, Jing Lu, Xuhui Fan, Qi Zhu, Hongcheng Sun

**Affiliations:** 1 Department of General Surgery, Shanghai General Hospital, Shanghai Jiao Tong University School of Medicine, Shanghai 200080, China; cdsdoctor@sjtu.edu.cn (D.C.); cxzxlych@126.com (Y.L.); lujing100@163.com (J.L.); zhuqiwish@sina.com (Q.Z.); 2 Department of Radiology, Shanghai General Hospital, Shanghai Jiao Tong University School of Medicine, Shanghai 200080, China; fanxuhui@sjtu.edu.cn

**Keywords:** hepatocellular carcinoma, transmembrane protein, prognosis, immune landscape, TMEM201

## Abstract

Aberrant transmembrane protein (TMEM) expression is implicated in tumor progression, but its functional role in hepatocellular carcinoma (HCC) is unclear. Thus, we aim to characterize the functional contributions of TMEM in HCC. In this study, four novel TMEM-family genes (TMEMs), TMEM106C, TMEM201, TMEM164, and TMEM45A, were screened to create a TMEMs signature. These candidate genes are distinguished between patients with varying survival statuses. High-risk HCC patients had a significantly worse prognosis and more advanced clinicopathological characteristics in both the training and validation groups. The GO and KEGG analyses unveiled that the TMEMs signature might play a crucial role in cell-cycle-relevant and immune-related pathways. We found that the high-risk patients had lower stromal scores and a more immunosuppressive tumor microenvironment with massive infiltration of macrophages and Treg cells, whereas the low-risk group had higher stromal scores and gamma delta T-cell infiltration. Moreover, the expression level of suppressive immune checkpoints increased as the TMEM-signature scores increased. Furthermore, the in vitro experiments validated TMEM201, one feature of the TMEMs signature, and facilitated HCC proliferation, survival, and migration. The TMEMs signature provided a more precise prognostic evaluation of HCC and reflected the immunological status of HCC. Of the TMEMs signature studied, TMEM201 was found to significantly promote HCC progression.

## 1. Introduction

Primary liver cancer has emerged as a leading cause of tumor death worldwide, ranking third among the main causes of cancer-related mortality. Hepatocellular carcinoma (HCC) accounts for approximately 85% of hepatocarcinomas, and it is refractory to nearly all currently available anti-tumor therapies [1]. Even for patients with early-stage HCC who are considered operative candidates, the 5-year survival rate for HCC remains low due to significant postsurgical recurrence and metastasis rates [2]. For patients with advanced HCC, treatment options are limited to systemic therapies with modest survival benefits (e.g., the combination of atezolizumab plus bevacizumab) [3]. Moreover, the heterogeneity of HCC complicates the clinical management of tumors, as not all patients respond to systemic therapies in the majority of clinical cases. Great variations in survival outcomes have been observed in patients with similar clinicopathological features [4]. Traditional tumor staging may be insufficient to predict prognoses for patients with HCC due to the complex pathogenic mechanism of the disease and the genetic background [5]. To address this growing and unmet medical need, it is essential to explore unknown mechanisms and identify novel prognostic indicators in HCC, thereby enabling the optimization of therapeutic strategies for personalized management.

Although little is known about their functional mechanism, accumulating evidence demonstrates the involvement of transmembrane proteins in tumorigenesis, which involves aberrant transmembrane protein (TMEM) expression in various cancers [6]. Several studies have demonstrated that elevated expressions of TMEM48 or TMEM97 are significantly associated with advanced tumor clinicopathological features, which contribute to a poor prognosis in non-small-cell lung cancer [7,8]. Similarly, TMEM17 can accelerate breast cancer (BRC) progression through the specific activation of the AKT/GSK3 pathway, indicating its potential as an oncological treatment target [9]. Additionally, it has been reported that transmembrane proteins, such as TMEM74 and TMEM106C, are essential for hepatocarcinogenesis [10,11]. However, in addition to its oncogenic functions, the TMEM exhibits tumor-suppressive properties. For instance, a high expression of TMEM25 in BRC is associated with favorable overall survival (OS), whereas high expressions of TMEM176A and TMEM173 in esophageal squamous cell carcinoma and HCC, respectively, are indicative of a favorable prognosis. In addition, increased TMEM expression probably promotes acquired chemoresistance [12,13,14]. The TMEM98 is closely associated with transarterial chemoembolization (TACE) refractoriness in HCC [15]. Notably, it is also characterized by immune-related properties involving T-helper 1 (Th1)-cell differentiation, indicating its capacity to regulate the tumor immune microenvironment (TIME) [16]. Overall, the exact mechanisms of TMEM in tumors remain uncertain and complex. An understanding of the role of TMEM in the tumor microenvironment (TME) would advance our understanding of the fundamental processes of tumor genesis and development, therefore revealing potential therapeutic intervention targets.

In this study, we gained a deeper understanding of the role of TMEM in HCC. Based on TMEM-family genes (TMEMs), we developed and validated the signatures of four genes, TMEM106C, TMEM201, TMEM164, and TMEM45A, for prognosis and risk classification in HCC patients. Given the results of the functional study of the TMEMs signature, both the prognostic value and the immunological characteristics affected by the TMEMs signature were comprehensively investigated. In addition, we evaluated TMEM201, a key factor in the TMEMs signature, as a representative feature to establish its function in hepatocarcinogenesis using in vitro studies. Our study provides a theoretical foundation for TMEMs’ role in HCC development and a valid biomarker, the TMEMs signature, for individualizing the management of HCC patients.

## 2. Results

### 2.1. Investigation of Differentially Expressed TMEMs

**Figure 1:**
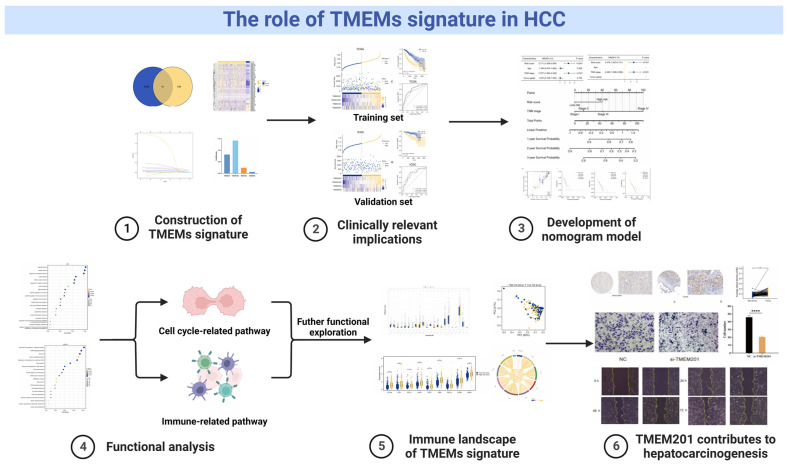
Flowchart portraying the present study.

**Figure 2:**
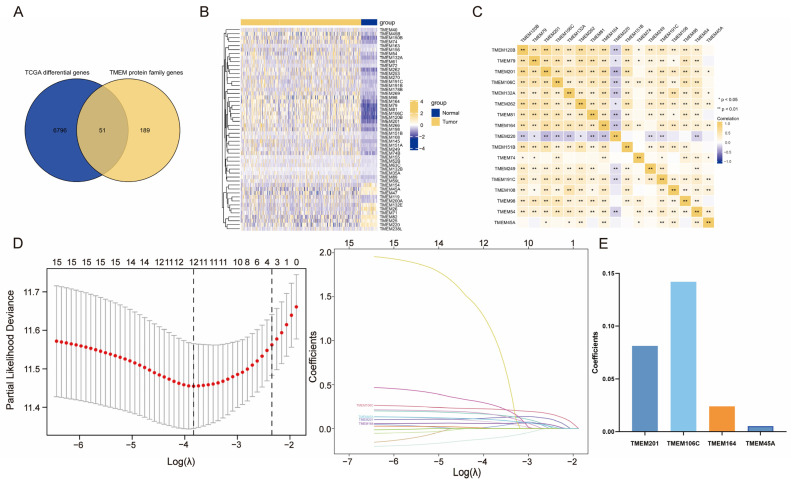
Construction of TMEMs signature. (**A**) Intersection of TMEMs and DEGs in HCC. (**B**) Heat map of 51 differentially expressed TMEMs. (**C**) Correlation analysis for candidate TMEM. (**D**) TMEMs signature filtered by the LASSO algorithm (Red dots: partial likelihood deviance; Different colored lines: the change of coefficients of each feature). (**E**) The LASSO coefficients of four features.

The study flowchart is depicted in Figure 1. A total of 327 well-defined TMEMs were included in this study. We explored the TMEMs’ expression profiles in both the normal and the HCC samples. Figure 2A,B summarize the TMEMs with significant differential expressions in HCC, of which only 17 were correlated with prognosis (Supplement Table S1) and all were closely correlated (Figure 2C). Based on these results, the LASSO algorithm was employed to screen out optimal prognostic TMEMs, identifying four candidate TMEMs for subsequent analysis (Figure 2D). The LASSO coefficient profiles of four features are depicted in Figure 2E.

### 2.2. Construction and Verification of TMEMs Signature

**Figure 3:**
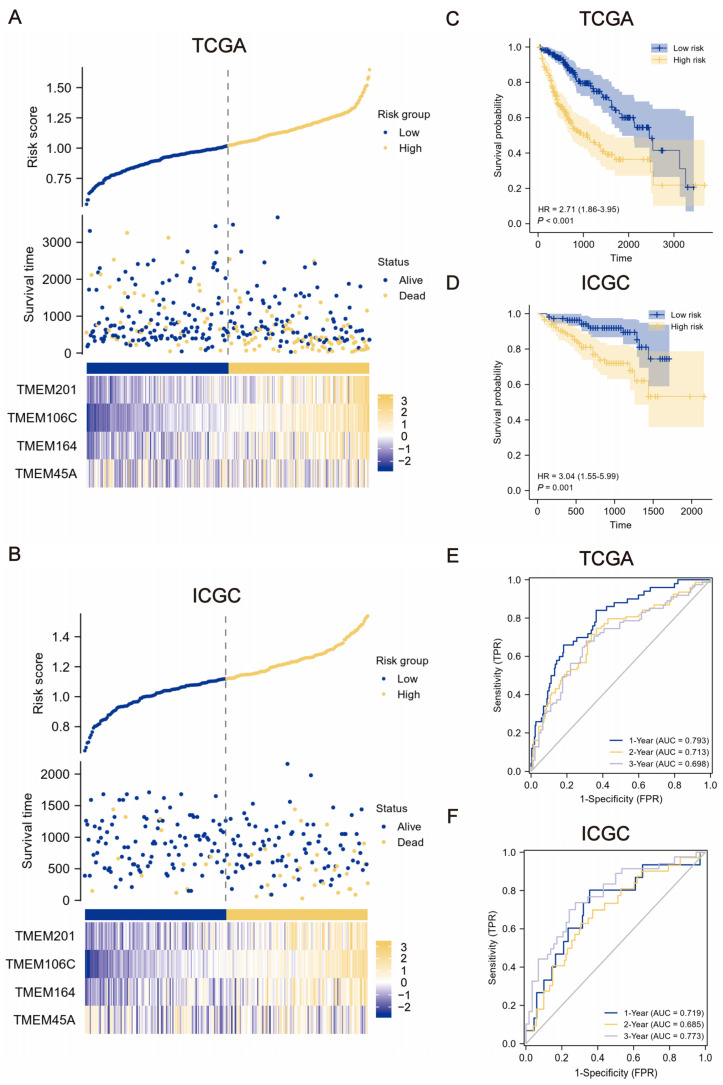
Identification and validation of TMEMs signature. (**A**,**B**) Distribution of the patients’ survival rates with varied TMEM signature scores and expression panel of four genes. (**C**,**D**) K–M curves of low- and high-risk groups. (**E**,**F**) Time-dependent ROC curves at 1-, 2-, and 3-year survival rates.

The TMEMs signature were constructed with LASSO coefficient-weighted expression levels using the following formula: TMEMs risk score = (0.1421 × TMEM106C) + (0.0813 × TMEM201) + (0.0240 × TMEM164) + (0.0054 × TMEM45A). The patients with TMEM scores below or above the median were categorized as low-risk and high-risk, respectively. As anticipated, the distributions of the patients’ survival with distinct scores and the heat maps of the expression levels of the four TMEMs revealed that the higher-risk patients had generally poorer survival, suggesting a predictive function for TMEMs signature in HCC (Figure 3A,B). Similarly, compared to the low-risk group, the K–M curves for the high-risk group demonstrated a clear tendency toward lower survival probabilities (*p* < 0.001, HR = 2.71 in the training set, Figure 3C; *p* = 0.001, HR = 3.04 in the validation cohort, Figure 3D). Furthermore, the time-dependent ROC analysis demonstrated that the TMEMs signature exhibited excellent prediction accuracy in a diverse and independent HCC set, with 1-, 2-, and 3-year area under the ROC curve (AUC) in TCGA = 0.793, 0.713, and 0.698, and in ICGC = 0.719, 0.685, and 0.773, respectively, compared with 0.500 for random predictions (Figure 3E,F).

### 2.3. Clinical Relevance of the TMEMs Signature

**Figure 4:**
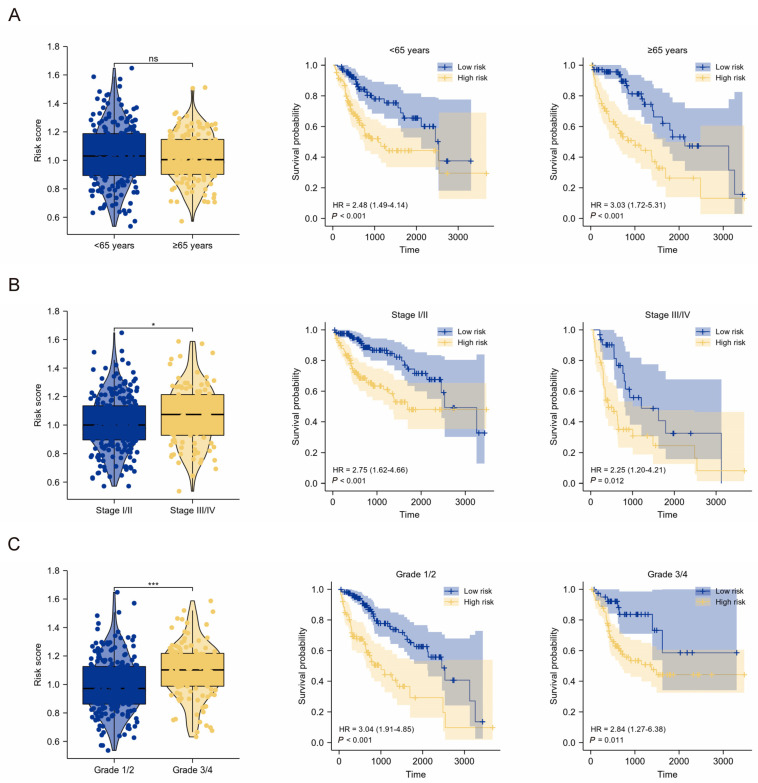
Stratified prognostic analysis of the TMEM signatures in the TCGA cohort. (**A**–**C**) The risk scores and K–M curves in clinical subgroups, containing (**A**) age subgroups, (**B**) stage subgroups, and (**C**) grade subgroups. *: *p* < 0.05, ***: *p* < 0.001, ns: *p* ≥ 0.05.

**Figure 5:**
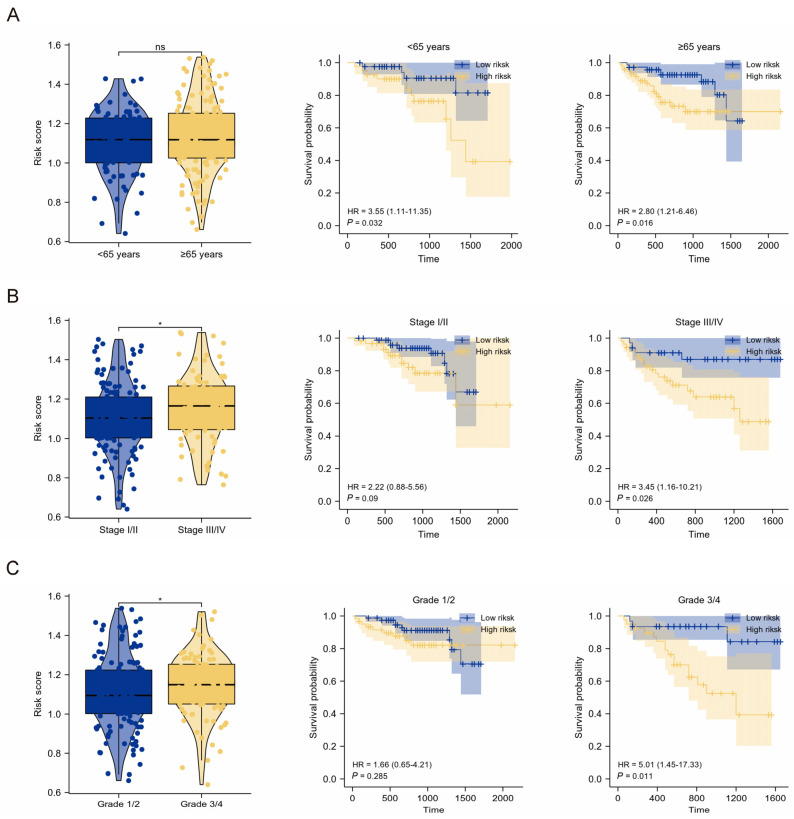
Stratified prognostic analysis of the TMEMs signature in the ICGC cohort. (**A**–**C**) The risk scores and K–M curves in clinical subgroups, containing (**A**) age subgroups, (**B**) stage subgroups, and (**C**) grade subgroups. *: *p* < 0.05, ns: *p ≥* 0.05.

To determine the clinical relevance of the TMEMs signature, the patients were grouped by clinical features and classified using the TMEMs signature. With the exception of the age groups, the TMEM risk-score distribution of the patients with diverse TNM stages and grades varied significantly in the TCGA cohort, indicating that a higher risk scores were predictive of poorer outcomes. In addition, the TMEMs signature exhibited excellent performance in the K–M subgroup analysis, in which the patients were stratified by age, stage, and grade (Figure 4A–C). Nonetheless, the ICGC cohort showed comparable results between the clinical features and the TMEM risk scores to those observed in the TCGA cohort, except for the absence of OS differences in the lower-stage/grade-HCC subgroup (Figure 5A–C).

### 2.4. Clinical Application of TMEMs Signature

**Figure 6:**
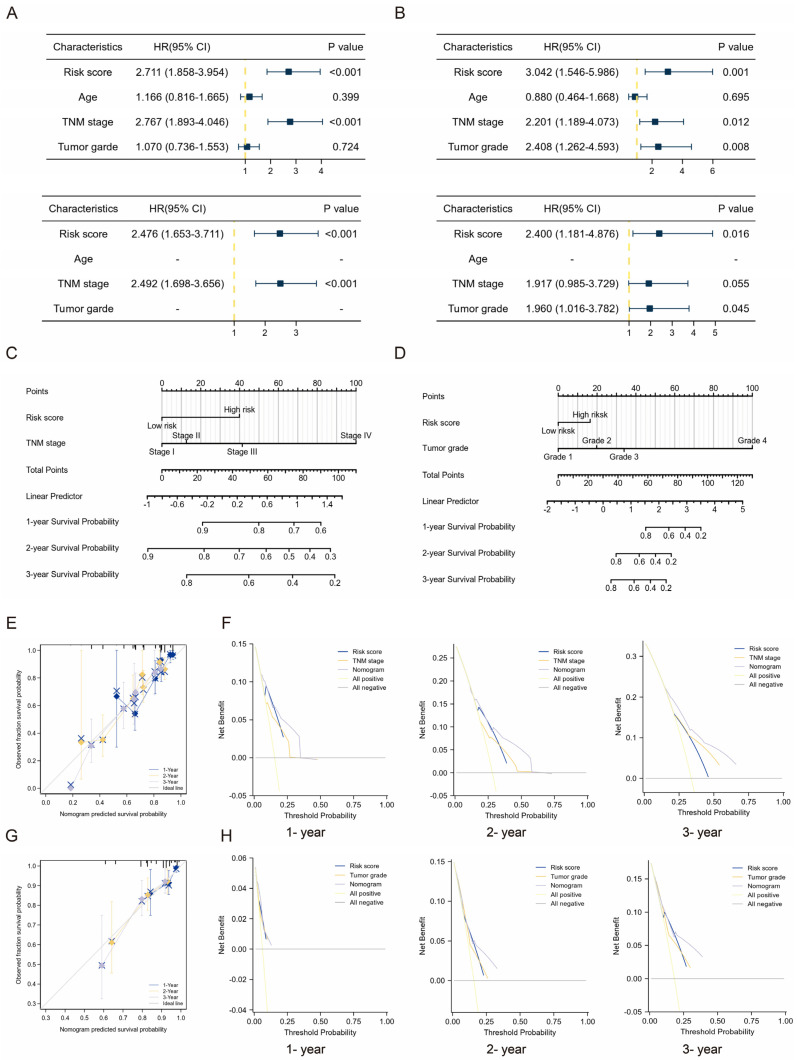
Development of a nomogram incorporating the TMEMs signature in combination with clinical variables. (**A**,**B**) Cox regression (univariate and multivariate) results of the risk variables used to develop (**C**,**D**) the nomogram, which were evaluated by (**E**,**G**) calibration curves and (**F**,**H**) DCA in the TCGA and ICGC cohorts, respectively.

As determined by Cox regression (the LIHC and LIRI sets, Figure 6A,B), the TMEMs scores were potential independent prognostic factor of HCC. Considering its clinical applications, we developed a nomogram incorporating TMEMs to facilitate prediction of OS (Figure 6C,D). There was a strong correlation between predicted survival and observed values in all cohorts (Figure 6E,G). DCA further indicated that our model has a greater clinical implementation significance than the tumor staging system (Figure 6F,H).

### 2.5. Functional Analyses Based on TMEMs Signature

**Figure 7:**
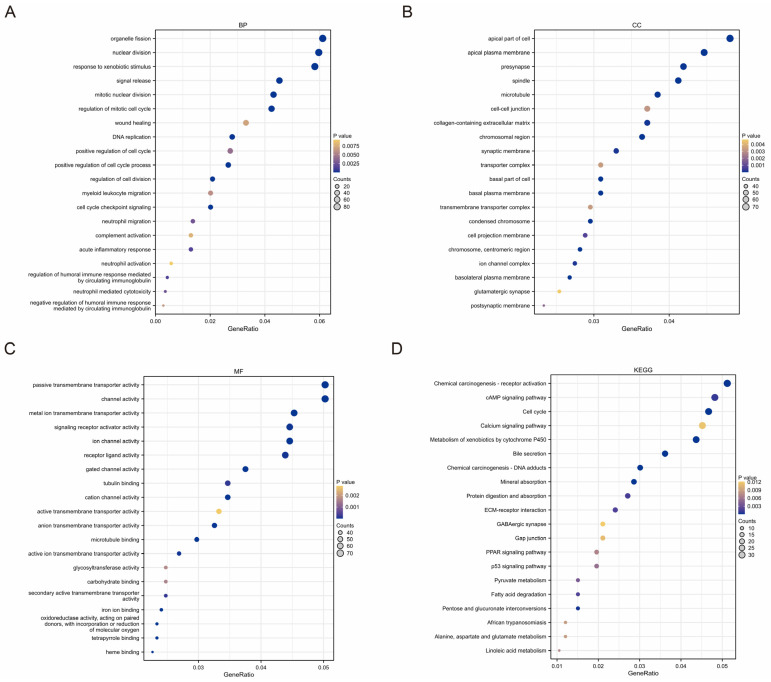
Functional analysis of TMEM signatures. Functional annotation of DEGs between low- and high-risk groups by GO and KEGG-pathway analyses. (**A**) BP: biological process; (**B**) CC: cellular compartment; (**C**) MF: molecular functions; (**D**) KEGG pathway.

According to the results above, the TMEMs signature were closely associated with HCC progression, but their exact function remained unknown. Thus, we conducted a GO classification and KEGG-pathway analyses based on the DEGs between the two groups. Notably, the functional analyses revealed that the TMEMs were predominantly associated with cell-cycle-related pathways and immune-related pathways, including nuclear division, wound healing, cell cycle, neutrophil migration, and acute inflammatory response (Figure 7A–D), implying the TMEMs’ prominent role in tumorigenesis and TIME in HCC.

### 2.6. HCC Immune Landscape Affected by TMEMs Signature

**Figure 8:**
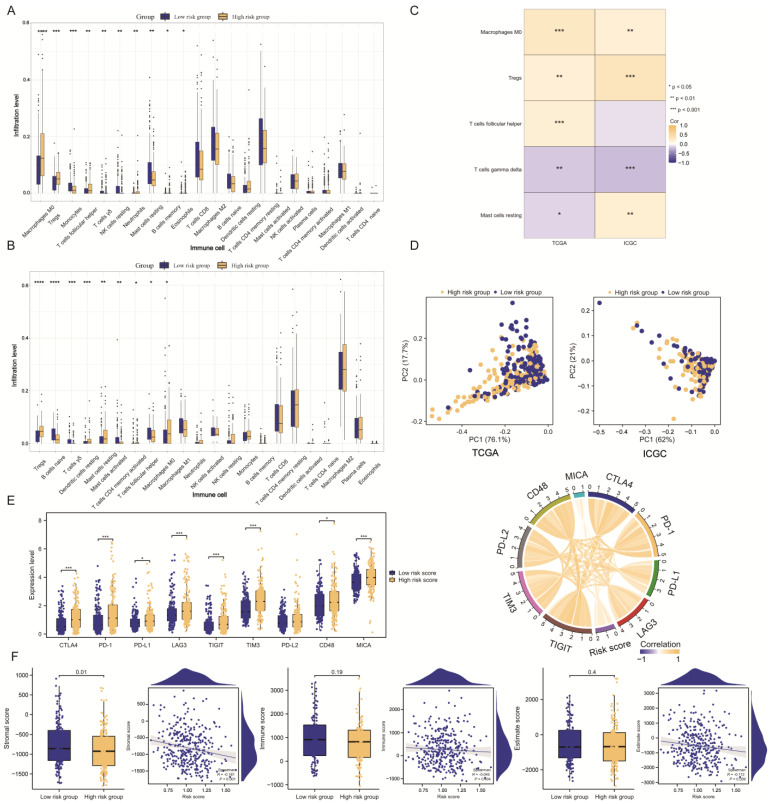
Landscape of the immune microenvironment in different risk groups stratified by TMEMs signature. Immunological landscapes in stratified risk groups: (**A**,**B**) Immune-cell landscape according to ‘CIBERSORT’ analysis. (**C**) Correlation of tumor-infiltrating immune-cell confirmed by two groups. (**D**) PCA based on immune cells. (**E**) Changes in immune-checkpoint expression. (**F**) Stromal score, immune score, and estimate score. *: *p* < 0.05, **: *p* < 0.01, ***: *p* < 0.001, ****: *p* < 0.0001.

Due to the prevalence of immune-related pathways, the ‘CIBERSORT’ algorithm was utilized to examine the amount of infiltrating immune cells in the HCC patients with varying risk scores. In the TCGA cohort, the proportion of suppressive immune cells, including macrophages and Tregs, increased as the risk scores of the TMEMs, resulting in the formation of an immunosuppressive TME. In contrast, the low-risk group exhibited a significantly higher amount of anti-tumor gamma delta T cells and resting NK cells (Figure 8A). Furthermore, the infiltration of risk-group-specific immune-cell subsets, such as macrophages, Tregs, and gamma delta T cells, was confirmed in the ICGC cohort (Figure 8B). Notably, there was a strong correlation between the TMEM scores and the primary immune cells (Figure 8C). The PCA further demonstrated the difference in infiltrating immune cells between the training and validation sets (Figure 8D).

Given the significance of immune-checkpoint inhibitors (ICIs) in TIME, we evaluated the changes in the TMEM scores in the various ICIs. The expression of ICIs (e.g., CTLA4, PD-1, PD-L1, LAG3, TIGIT, TIM3, CD48, and MICA) increased in conjunction with the risk score, demonstrating a positive connection (Figure 8E). The patients with higher TMEM scores appeared to be more susceptible to ICIs, and the combined TMEM scores may have functioned as essential immunotherapy-predictive markers. Using the ESTIMATE method, the lower-risk patients were found to have considerably higher stromal scores, which were correlated with improvements (Figure 8F). Our results revealed the role of TMEMs in TIME, confirming the findings of previous functional analyses.

### 2.7. TMEM201 in HCC Development

**Figure 9:**
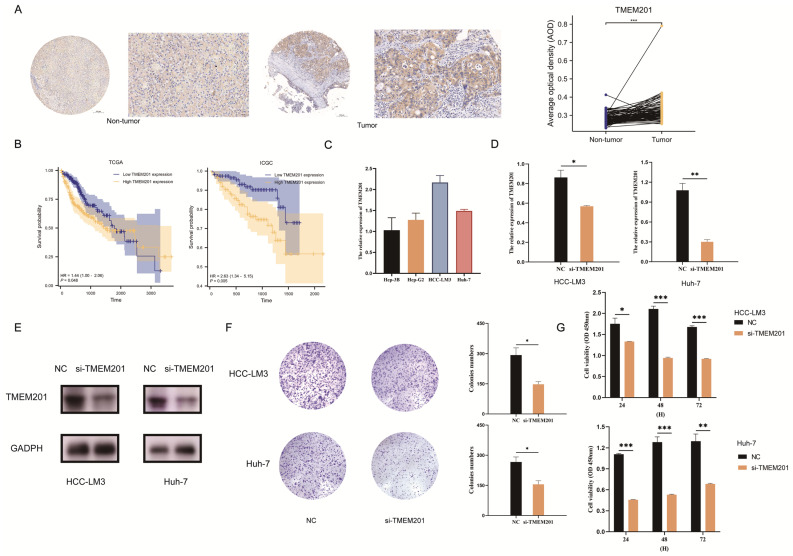
Identification of the role of TMEM201 in HCC. (**A**) TMEM201 expression in HCC TAM (5× and 20×). (**B**) Differences in OS between high-/low-TMEM201-expression groups. (**C**) TMEM201 expression in different cell lines according to qPCR. (**D**) The efficiency of siRNA targeting TMEM201. (**E**) The protein levels of TMEM201 in si-TMEM201 cell lines according to WB. (**F**,**G**) Colony-formation assay and CCK-8 assay for TMEM201-knockdown-cell lines. *: *p* < 0.05, **: *p* < 0.01, ***: *p* < 0.001.

**Figure 10:**
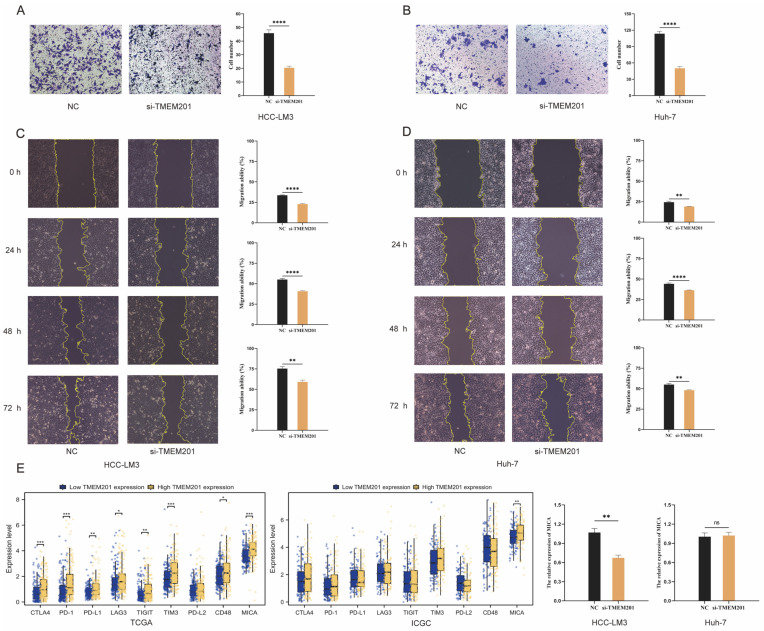
Identification of the role of TMEM201 in HCC (**A**,**B**). Transwell assay for TMEM201 knockdown cell lines (10×). (**C**,**D**) Wound-healing in si-TMEM201 cell lines (10×). (**E**) The effect of TMEM201 on the expression of ICIs. *: *p* < 0.05, **: *p*< 0.01, ***: *p* < 0.001, ****: *p* < 0.0001, ns: *p* ≥ 0.05.

In this study, the cell-cycle-related pathways represented additional important functions of the TMEMs. A representative member of the TMEMs’ signature, TMEM201, was highly expressed in HCC from tissue microarrays (TAM) (Figure 9A). The up-expressed TMEM201 contributed to lower OS in two cohorts (Figure 9B). The RT–qPCR analysis validated the expressed TMEM201 level in the hepatoma cell lines (Figure 9C). To evaluate the function of TMEM201, the si-TMEM201 hepatoma cell lines were constructed (Figure 9D), and a decrease in TMEM201 protein expression was found (Figure 9E). In both the colony formation and the CCK-8 assay, the si-TMEM201 cell lines’ proliferative capacity was significantly reduced (Figure 9F,G). In addition, the reduction in TMEM201 expression decreased the migratory capacity of the HCC-LM3 and Huh-7 cell lines in the transwell assay (Figure 10A,B) and wound-healing assay (Figure 10C,D). All the results indicated that TMEM201 may have a significant impact on the advancement of HCC. Furthermore, both cohorts demonstrated a significant relationship between the high expression of TMEM201 and the increased expression of MICA, which was confirmed in the si-TMEM201 hepatoma cell lines (Figure 10E).

## 3. Discussion

Hepatocellular carcinoma is one of the most refractory malignancies, and it is characterized by rapid progression, high recurrence, and heterogeneity. However, the hepatocarcinogenetic mechanism remains unknown, leading to poor prognoses [2]. The exploration of the pathogenesis of HCC drives the development of innovative therapeutic approaches [17]. For instance, the combination of TACE, nivolumab (anti-PD1 inhibitor), and molecular targeted therapies significantly improves the OS (median OS, 19.2 months) for advanced HCC, with a high objective response rate, of 60.1% [18]. In addition, given the heterogeneity of HCC patients, combining multiple markers for the risk stratification and management of HCC is of great value. Previous studies demonstrated the oncogenic role of the TMEM protein in various cancers, such as BRC and colorectal cancer [19]. However, no comprehensive analyses of TMEMs in HCC have been reported. Herein, we elucidated the role of TMEMs in HCC, developed a novel prognostic signature, and explored the relevance of TMEMs to TIME, which may provide insight into the optimal treatment strategy.

In this study, we extracted 17 candidate TMEMs with distinct prognostic implications in HCC from the TCGA cohort. We further constructed the TMEM signatures using LASSO regression. This signatures included four risk factors for the prediction of OS in HCC, TMEM106C, TMEM201, TMEM164, and TMEM45A. Nevertheless, there are limited data on the role of these genes, and only TMEM106C and TMEM45A have been described in HCC. For instance, TMEM106C was overexpressed in hepatoma tissues and strongly correlated with a poor prognosis, suggesting its involvement in the occurrence and progression of liver cancer [10]. Moreover, LINC0023 regulates its cancer-promoting effects through the apoptosis pathway [20]. Regarding TMEM45A, HCC chemoresistance can be regulated by TMEM45A, although the underlying mechanism is still unknown [21]. For other risk factors, TMEM201 is a positive modulator that can activate TGF-β/SMAD2/3 signaling to promote BRC metastasis [22]. In addition, TMEM164 loss attenuates the anti-tumor response of ferroptosis-induced cytotoxicity [23]. Up-expressed TMEM164 in pancreatic cancer may improve survival and reshape TIME [23]. Collectively, prior studies demonstrated that TMEMs exert a significant effect on tumor development.

Notably, the novel TMEMs signature exhibited excellent prognostic ability in both the training and the validation sets. According to the K–M and time-dependent ROC curves, the HCC patients with poor outcomes were screened out promptly. In addition, our findings elucidate the clinical relevance of the varying risk scores of patients. As anticipated, the HCC patients with a higher stage or grade had a higher risk score. In the K–M subgroup analysis, the TMEM-based risk stratification also exhibited good performance in predicting OS. Using multivariate Cox analysis in two sets, we validated the TMEMs signature as an independent prognostic indicator for HCC. To expedite its application in clinical settings, we developed an innovative nomogram integrating TMEM scores and clinical features for the personalized management of HCC. These TMEM-based nomograms were superior to the current tumor-staging system, demonstrating excellent accuracy and applicability in predicting OS. Overall, these findings suggested that the TMEMs signature showed a favorable prognostic value.

Few studies have been conducted on the functional properties of TMEMs. The GO and KEGG annotations provided insights into the possible roles of TMEMs in HCC. The DEGs in the low- and high-risk groups were primarily enriched in pathways associated with cell proliferation or immunity, which emerged as crucial regulatory pathways in the modification of the TME. Consequently, we further investigated the relationship between the TMEMs signature and the TIME in HCC. As demonstrated by our CIBERSORT analysis, several key immune-cell types, including macrophages, Tregs, and gamma delta T cells, were closely correlated with the TMEM scores. Specifically, the high-risk group was characterized by the increased infiltration of immune cells, such as macrophages and Tregs, which corresponds to the definition of a “cold tumor” phenotype [24], whereas the low-risk group was characterized by the presence of gamma delta T cells to activate the anti-tumor response. These observations implied that TMEMs might regulate the phenotypic plasticity of the TME to form hot or cold tumors. The PCA also revealed that the distribution of the immune-cell subtypes was distinct in the two risk groups. Similarly, these trends were apparent in the ‘ESTIMATE’ algorithm of the stromal score. Therefore, TMEMs can influence the immunological landscapes of HCC, leading to different prognoses.

Regarding the cell-cycle function, the role of TMEM201, a factor in the TMEMs signature, was initially identified in HCC. The si-TMEM201 HCC cell lines expressed TMEM201 at a lower level compared to the NC group, demonstrating the cancer-promoting properties of TMEM201. The patients with lower TMEM201 expression had a longer OS with HCC in both the training and the validation set. The potential of si-TMEM201 to inhibit hepatocarcinogenesis was subsequently validated in the hepatoma cell lines. Moreover, high TMEM201 levels were significantly correlated with increased MICA expression, which plays a role in tumor-immune-escape mechanisms. Due to its expression in various types of tumor, MICA presents an appealing target for immunotherapy [25]. All the in vitro studies revealed that TMEM201 may promote the progression of HCC.

## 4. Methods and Materials

### 4.1. Data Sources

The Cancer Genome Atlas database (TCGA)-LIHC cohort was obtained as the training set [26]. For the validation set, the International Cancer Genome Consortium (ICGC)-LIRI-JP cohort was collected [27]. In this study, expressed genes with raw read counts in fewer than half of the samples were excluded. Patients with incomplete clinical information, survival time less than 30 days, repeated sequencing, or missing values were excluded. Moreover, the list of genes in the TMEM family was downloaded from the HUGO Gene Nomenclature Committee (HGNC).

### 4.2. Identification of TMEMs in HCC

Differentially expressed genes (DEGs) were assessed. The |logFC| > 1 with *p* < 0.05 were utilized as the screening cutoff to detect significant DEGs in the training set. The 327 TMEMs taken from the HGNC database and the DEGs were intersected by Venn diagrams, of which the distribution was presented by heatmap.

### 4.3. Construction and Validation of TMEMs Signature

A Univariate Cox assay was applied to identify the univariate prognostic features, with *p* < 0.05. Next, optimal features were selected through LASSO regression (10-fold cross-validation). We then used the LASSO coefficients profiles of candidate TMEMs to estimate risk scores for each patient using the detailed formula shown below: TMEMs risk score (patient) = ∑ (coefficient I × expression of gene i) [28]. For both training and validation sets, risk-stratification groups were then created based on TMEM-risk-score median, including low- and high-risk groups. Subsequently, we plotted patients’ survival distributions with various TMEM risk scores and the expressed patterns of TMEM signatures to determine the association between the signature score and HCC prognosis. Kaplan–Meier (K–M) curves were created for survival comparison in risk-stratified groups, and receiver operating characteristic (ROC) curves (time-dependent) were generated to assess the discriminative power of the TMEMs-signature risk model. In addition, the clinicopathological relevance of TMEMs signature was explored by testing the difference in risk score between subgroups classified by patient features. Furthermore, we used Cox regression (univariate and multivariate) to characterize whether TMEM risk score was an independent prognostic indicator affecting OS. Next, the corresponding novel nomogram was plotted and its clinical application was estimated by decision-curve analysis (DCA) and calibration curves.

### 4.4. Functional Enrichment Analysis

To better uncover the possible mechanisms linking TMEMs in HCC, DEGs between two groups (low scores vs. high scores) were identified. Gene Ontology (GO), which was comprised of biological processes (BP) and cellular components (CC), as well as molecular functions (MF), and KEGG enrichment analyses of the significant DEGs described above were investigated.

### 4.5. Immune Landscapes of TMEMs Signature

To quantify immune-cell infiltration in two groups (low-risk vs. high-risk), we performed CIBERSORT R scripts to refine immune-cell subsets in individual HCC samples from the TCGA and ICGC cohorts. The correlation of the primary infiltrating immune cells was also established. Simultaneously, based on the 22 immune cells from CIBERSORT algorithms, we applied principal component analysis (PCA) to estimate the capability of differentially infiltrated immune cells to separate risk-stratification groups. Furthermore, the relationship between TMEM risk score and biomarkers of immunotherapies, such as immune checkpoints, was evaluated in the low- and high-risk groups. Stromal scores and immune scores of HCC samples were calculated via the ESTIMATE R package. Furthermore, the effect of TMEM201 on the expression of ICIs was evaluated.

### 4.6. Cell Culture and Transfection

Four human hepatoma cell lines, including HCC-LM3, Hep-3B, Huh-7, and Hep-G2 were obtained from the cell bank at the Chinese Academy of Sciences (Shanghai, China) and maintained under conventional culture conditions. The siRNA targeting TMEM201 and si-negative control (NC) listed in Supplementary Table S2 were constructed by Shanghai Gene Pharma, and transfection was conducted with Lipofectamine 2000 reagent from Invitrogen (Thermo Fisher Scientific, Waltham, MA, USA), based on the manufacturer’s protocol.

For TMEM201-expression analysis, total RNA and protein were extracted from each cell line. Synthesized cDNA carried out with the reverse-transcription kit from EnzyArtisan (Shanghai, China) were further determined by quantitative real-time PCR (qRT-PCR) with 2 × S6 Universal SYBR qPCR Mix from EnzyArtisan (Shanghai, China). The protein expression of TMEM201 was then evaluated by Western blotting (WB). The GAPDH was used as the internal reference. Signals were developed by ECL reagent from Thermo Fisher (USA). All primer sequences for qPCR and primary and secondary antibodies for WB are listed in Supplementary Table S2.

### 4.7. Immunohistochemistry (IHC) for Tissue Microarrays (TAM)

Ninety cases of tumor samples and matched adjacent tissues were collected from HCC patients at Shanghai General Hospital, China. Anti-TMEM201 from Proteintech (#24092-1-AP) was used as the primary antibody for IHC on TAM. The average optical density (AOD) was automatically determined using Servicebio [29]. The relative expression of TMEM201 was calculated by comparing the AOD values between cancerous and adjacent tissues.

### 4.8. Assessment of Cell Proliferation and Evaluation of Cell Migration

Cells were seeded in a 96-well plate for cell-counting kit-8 (CCK-8) assay at three timepoints (24 h, 48 h, and 72 h). After adding the CCK-8 reagent (Dojindo Laboratories, Kumamoto, Japan) and incubating for 1 h, optical density at a 450 nm wavelength (OD450 nm) was measured. Additionally, hepatoma cells were cultivated in 6-well culture plates for colony-formation assay. When the colony was formed and fixed with 4% methanol, cells were photographed to quantify the number of clones after staining with crystal-violet dye.

Hepatoma cells were grown in a 6-well plate until approximately 90% confluence, at which time scratch wounds were generated by sterile pipette tips. Following removal of suspended cells, the scratch wound was observed and photographed by a phase-contrast microscope at 0 h, 24 h, 48 h, and 72 h time points. For transwell assay, 600 μL complete medium was filled in the bottom well, while cells were plated in the top chamber in 200 μL serum-free medium. After 24 h, cells on the membranes were preserved with 4% methanol, dyed with crystal violet, and photographed under microscopic fields.

### 4.9. Statistical Analysis

Data from cohorts were measured on the R-4.1.1 platform, with experimental data analyzed in GraphPad Prism 9.0. For statistical comparisons, Wilcoxon signed-rank test or Student *t*-test were performed. With the Spearman method, correlation analyses were performed. Experiments were independently performed and repeated at least 3 times. Statistical significance was assigned at a *p*-value of less than 0.05 (*p* < 0.0001: quadruple asterisks, *p* < 0.001: triple asterisks, *p* < 0.01: double asterisks, *p* < 0.05: asterisk, and *p* ≥ 0.05: ns).

## 5. Conclusions

In conclusion, our findings yielded novel insights regarding the function of TMEMs in HCC. The TMEMs signature and the corresponding nomogram enabled the risk stratification of HCC patients for personalized treatments, demonstrating potential prognostic value. Based on the pathway-analysis results, the TMEMs signature had strong correlations with immune-cell infiltration and the identification of hot tumors and cold tumors. In addition, the function of TMEM201, a feature of the TMEMs signature, was verified for the first time in hepatoma cells. Our study presented a detailed examination of the clinical relevance and immune landscapes of the TMEMs signature in HCC.

## Data Availability

All relevant data presented by this article are available from the corresponding author up on reasonable requests.

## Supplementary Materials

The following supporting information can be downloaded at: https://www.mdpi.com/article/10.3390/ijms241210285/s1.
