# Supplementary material for: Characterization of the Clinical Significance and Immunological Landscapes of a Novel TMEMs Signature in Hepatocellular Carcinoma and the Contribution of TMEM201 to Hepatocarcinogenesis
Source: Int J Mol Sci. 2023 Jun 17;24(12):10285. doi: 10.3390/ijms241210285 (PMC10299404; doi:10.3390/ijms241210285)
Supplement: Supplementary file 1 [file ijms-24-10285-s001.zip › ijms-2378391-supplementary/Supplement Table S1.pdf]

Table S1. Univariate Cox regression of candidate TMEMs

TMEM protein family genes	HR (95% CI)	P value
TMEM120B	2.066 (1.451-2.941)	<0.001
TMEM79	1.838 (1.352-2.500)	<0.001
TMEM145	1.069 (0.876-1.304)	0.510
TMEM26	1.711 (0.985-2.971)	0.056
TMEM201	1.760 (1.395-2.221)	<0.001
TMEM106C	1.599 (1.330-1.924)	<0.001
TMEM132A	1.171 (1.044-1.312)	0.007
TMEM262	2.891 (1.621-5.157)	<0.001
TMEM81	1.739 (1.305-2.319)	<0.001
TMEM150B	1.103 (0.982-1.239)	0.099
TMEM164	1.566 (1.289-1.903)	<0.001
TMEM220	0.797 (0.687-0.925)	0.003
TMEM266	1.186 (0.916-1.535)	0.195
TMEM198	1.035 (0.870-1.232)	0.696
TMEM178B	1.124 (0.876-1.442)	0.357
TMEM253	0.972 (0.508-1.858)	0.932
TMEM71	0.774 (0.476-1.261)	0.304
TMEM151B	83.395 (6.315-1101.307)	<0.001
TMEM74	1.347 (1.086-1.671)	0.007
TMEM61	1.146 (0.968-1.358)	0.114
TMEM154	0.922 (0.692-1.228)	0.577
TMEM249	2.037 (1.057-3.926)	0.034
TMEM74B	1.163 (0.999-1.354)	0.052
TMEM191C	2.967 (1.241-7.094)	0.014
TMEM108	2.552 (1.384-4.706)	0.003
TMEM98	1.190 (1.048-1.351)	0.007
TMEM82	0.929 (0.846-1.020)	0.122
TMEM151A	1.091 (0.952-1.249)	0.209
TMEM132B	1.277 (0.833-1.957)	0.262
TMEM119	1.050 (0.926-1.191)	0.446
TMEM163	1.072 (0.922-1.246)	0.364
TMEM47	0.986 (0.850-1.144)	0.853
TMEM40	1.246 (0.924-1.681)	0.150
TMEM132E	0.783 (0.502-1.220)	0.279
TMEM155	1.346 (0.958-1.891)	0.087
TMEM52B	0.967 (0.710-1.317)	0.831
TMEM270	1.066 (0.858-1.326)	0.564
TMEM72	1.193 (0.997-1.429)	0.054
TMEM25	0.999 (0.848-1.176)	0.987

TMEM269	1.800 (0.620-5.227)	0.280
TMEM54	1.188 (1.065-1.325)	0.002
TMEM191B	0.978 (0.576-1.660)	0.934
TMEM89	1.367 (0.640-2.921)	0.420
TMEM45A	1.184 (1.072-1.307)	<0.001
TMEM63C	1.243 (0.956-1.615)	0.104
TMEM59L	1.234 (0.860-1.771)	0.253
TMEM45B	1.050 (0.956-1.153)	0.307
TMEM200A	1.176 (0.906-1.525)	0.223
TMEM35A	1.209 (0.819-1.786)	0.340
TMEM238L	1.001 (0.666-1.502)	0.998
TMEM156	1.112 (0.997-1.241)	0.057
