# Supplementary material for: Characterization of the Clinical Significance and Immunological Landscapes of a Novel TMEMs Signature in Hepatocellular Carcinoma and the Contribution of TMEM201 to Hepatocarcinogenesis
Source: Int J Mol Sci. 2023 Jun 17;24(12):10285. doi: 10.3390/ijms241210285 (PMC10299404; doi:10.3390/ijms241210285)
Supplement: Supplementary file 1 [file ijms-24-10285-s001.zip › ijms-2378391-supplementary/Supplement Table S2.pdf]

Table S2.

The primers sequences information of the genes and siRNA used in our study

Symbol	Sequences
**TMEM201**	F AGGATACGCTGGTGCCCTATG<br>R TCTCCTGGAAGCCGTTGTACTG
**MICA**	F- CTCGGATTTCAGCCTCTGATG<br>R- CAGGAAACTGAGGCACCAAGAG
**GADPH**	F AAGGTGAAGGTCGGAGTCAAC<br>R GGGGTCATTGATGGCAACAATA
**si-TMEM201**	Sense 5'-3' ACCAGACCACCAAGAUCAATT<br>Antisense 5'-3' UUGAUCUUGGUGGUCUGGUTT
**si-NC**	Sense 5-3' UUCUCCGAACGUGUCACGUTT-3′<br>Antisense 5-3' ACGUGACACGUUCGGAGAATT-3′

The primary and secondary antibodies for Western blotting

**Anti-TMEM201**	Proteintech , #24092-1-AP
**Anti-GADPH**	CST, #97166
**Anti-rabbit IgG-HRP-linked Antibody**	CST, #7074
**Anti-mouse IgG-HRP-linked Antibody**	CST, #7076
